# Subcutaneous Immunization with Fusion Protein DnaJ-ΔA146Ply without Additional Adjuvants Induces both Humoral and Cellular Immunity against Pneumococcal Infection Partially Depending on TLR4

**DOI:** 10.3389/fimmu.2017.00686

**Published:** 2017-06-12

**Authors:** Yufeng Su, Dagen Li, Yan Xing, Hong Wang, Jian Wang, Jun Yuan, Xiaofang Wang, Fang Cui, Yibing Yin, Xuemei Zhang

**Affiliations:** ^1^Department of Laboratory Medicine, Key Laboratory of Diagnostic Medicine (Ministry of Education), Chongqing Medical University, Chongqing, China; ^2^Department of Laboratory Medicine, People’s Hospital of Changshou, Chongqing, China

**Keywords:** *Streptococcus pneumoniae*, DnaJ-ΔA146Ply, immune response, adjuvant, TLR4

## Abstract

Subunit vaccines that are poorly immunogenic are often combined with adjuvants for immunization. Our previous research identified a pneumolysin variant (ΔA146Ply), a Toll-like receptor 4 agonist, that was an effective adjuvant in the protection of fusion protein DnaJ-ΔA146Ply against mucosal *Streptococcus pneumoniae* infections. For pneumococcal vaccines, World Health Organization recommend injection as a regular vaccination approach. Subcutaneous immunization is a common and effective method of injection, so we explored the immunity mechanism of subcutaneous immunization with DnaJ-ΔA146Ply. We found that mice immunized subcutaneously with fusion proteins ΔA146Ply-DnaJ and DnaJ-ΔA146Ply produced a higher anti-DnaJ IgG titer than when DnaJ alone was administered. DnaJ-ΔA146Ply induced both B-cell and T-cell-dependent protection against both colonization and lethal pneumococcal infections. Levels of IFN-γ, IL-4, and IL-17A were also elevated in DnaJ-ΔA146Ply immunized mice. However, all these effects were negated in TLR4^−/−^ mice compared to WT mice immunized with DnaJ-ΔA146Ply. B-cell-deficient μMT mice, nude mice, IFN-γ^−/−^, and IL-4^−/−^ mice immunized with DnaJ-ΔA146Ply could not resist infection with pneumococci. IL-17A^−/−^ and TLR4^−/−^ mice did not benefit from DnaJ-ΔPly immunization in colonization experiments although their survival was not impaired compared with WT mice. Collectively, our data indicated that ΔA146Ply can be a potential subcutaneous adjuvant, and the DnaJ-ΔA146Ply fusion protein induces both humoral and cellular immune response to resist *S. pneumoniae* infection. The protective effect of colonization also depends on TLR4.

## Introduction

*Streptococcus pneumoniae* is an important human pathogen that can causes fatal infections including bacterial pneumonia and meningitis, and the less severe, but common, sinusitis, and acute otitis media (1, 2). *S. pneumoniae* infections in children have a 16% mortality rate according to the World Health Organization and vaccination is the most effective method for prevention (3). There are capsular polysaccharide-based vaccines available but these show weak effects in infants and have a limited serotype coverage so serotype conversion limits their use (3–5). Infant morbidity caused by non-capsular strains is increasing so capsule-based vaccines are not an alternative.

Protein vaccines are a new type of subunit vaccine prepared by genetic engineering technology that are able to induce T-cell-dependent protection and are economical and easy to produce. Protein vaccines for *S. pneumoniae* infections have completed phase I and II clinical trials and include IC-47, PspA, PlyD1, and PhTD (6).

The heat shock protein DnaJ (HSP 40) and pneumolysin (Ply) are important virulence factors of *S. pneumoniae* and highly conserved among serotypes (7, 8). Both DnaJ and a Ply deletion mutant protein (ΔA146Ply) are effective candidate protein vaccines against pneumococcal infections and are minimally toxic (6–9). We have observed that ΔA146Ply has mucosal adjuvant capacity and mouse immunization with a DnaJ-ΔA146Ply fusion protein through the mucosa-induced high level protection in the absence of an adjuvant (10).

*Streptococcus pneumoniae* is a common colonizing bacterium of the respiratory tract in humans, especially in children under 2 years old (11). However, it is hard for children to accurately control the dose of vaccine when vaccinated through the mucosa. And mucosal immunization would induce immune tolerance. In fact, World Health Organization’s “Target Product Profile” presently prefers *Streptococcal* vaccines to be given by injection (12). For subcutaneous immunization, antigen is slower to absorb compared with intramuscular immunization, helping to prolong the stimulation of host (13, 14). Therefore, the purpose of this study was to explore whether ΔA146Ply play a role of a subcutaneous immune adjuvant, prompting the fusion protein DnaJ-ΔA146Ply to induce the host to produce a much better immune protection against pneumococcal infections.

Vaccine protection relies on both humoral and cellular immunity although they have separate roles (8, 10, 13, 15). For instance, vaccines based on capsular antigens primarily depend on humoral immunity while whole-cell vaccines induce humoral and cellular responses and their protective effects against colonization depends on CD4^+^ Th17 cells (14–16). Adjuvanted protein vaccines introduced *via* the mucosal or subcutaneous routes induce humoral and cellular immunity. However, protection against invasive infections or colonization varies with the immunizing protein and pathway (17–19). For example, PspA5 can induce systemic IgG antibodies to high titers to against invasive infection (18). Our previous data showed mucosal immunization with DnaJ-ΔA146Ply fusion protein induces both mucosal and systemic immunity (10). It was probably expected that subcutaneous immunization would increase systemic immunity. In this study, we explored the immune mechanism of subcutaneous immunization with DnaJ-ΔA146Ply.

Pattern recognition receptors such as the toll-like receptors (TLRs) present on immune and epithelial cells recognize microorganisms and prime the immune response (20–22). Detection of microbes by TLRs is an important step in activation of innate responses and is critical for robust adaptive immune responses. TLR2 and TLR4 are primarily involved in recognizing and initiating a signaling response to bacterial components including responses to *S. pneumoniae* vaccines (20, 22). DnaJ and Ply are the components of DnaJ-ΔA146Ply fusion protein and both are TLR4 ligands (20, 21). In the current study, we identified TLR4 contributions in immune protection elicited by DnaJ-ΔA146Ply vaccination.

The results obtained in this study show that DnaJ-ΔA146Ply is a promising subcutaneous protein vaccine and that ΔA146Ply acts as a subcutaneous immune adjuvant. TLR4 affects the strength of cellular and humoral immunity, thus contributing to host protection against *S. pneumoniae* infections. These findings could contribute to the accumulating data on the use of subcutaneous protein vaccines.

## Materials and Methods

### Mice

Specific pathogen-free, female and male C57BL/6, and BALB/c wild-type mice (6–8 weeks old) were purchased from Beijing HFK Biotechnology, and raised at Chongqing Medical University, Chongqing, China. B-cell-deficient BALB/c mice (μMT mice) were obtained from the Chinese Academy of Sciences, China. Nude (*nu/nu*) mice were purchased from Beijing HFK Biotechnology. IFN-γ-deficient C57BL/6 mice (B6.129S7-Ifng^tmlTs^/J) were purchased from the Model Animal Research Center of Nanjing University, Nanjing, China. IL-4-deficient C57BL/6 mice (B6.129P2-Il4^tmlCgn^/J) and IL-17A-deficient C57BL/6 mice were obtained from the Jackson Laboratory (Bar Harbor, ME, USA). All animal experiments were performed under the guidance of the Institutional Animal Care and Use Committee of Chongqing Medical University.

### Bacterial Strains and Growth Conditions

*Escherichia coli* strains BL21-DnaJ, BL21-ΔA146Ply, BL21-DnaJ-ΔA146Ply, and BL21-ΔA146Ply-DnaJ have been constructed previously in our laboratory (8, 10). *E. coli* strains were grown in Luria–Bertani medium at 37°C. *S. pneumoniae* strain D39 (NCTC7466, serotype 2) was obtained from the National Collection of Type Culture (London, UK). Clinical isolates of *S. pneumoniae* 19F (CMCC 31693) and 6B (CMCC 31207) were purchased from the National Center for Medical Culture Collections (Beijing, China). All *S. pneumoniae* strains were cultured on Columbia-Nalidixic Acid (CNA) plates containing 5% sheep blood or in Casitone-Yeast extract (C + Y) medium both in an atmosphere of 5% CO_2_ at 37°C.

### Expression and Purification of Recombinant Proteins

Recombinant proteins ΔA146Ply, DnaJ-ΔA146Ply, and ΔA146Ply-DnaJ were purified after IPTG (isopropyl β-d-1-thiogalactopyranoside) induction using standard protocols (10). IPTG induction conditions differed and were as follows: DnaJ, 0.2 mM IPTG, 20°C, 130 r/min, 8–10 h induction; ΔA146Ply 0.5 mM IPTG, 20°C, 130 r/min, 8 h induction; DnaJ-ΔA146Ply 0.01 mM IPTG, 15°C, 120 r/min, 8 h induction; ΔA146Ply-DnaJ 0.1 mM IPTG, 20°C, 130 r/min, 8 h induction. The proteins were purified with commercial Ni^2+^ chromatography columns using procedures recommended by the manufacturer (GE Healthcare, Little Chalfont, UK). Column fractions were analyzed by 10% SDS-PAGE. Proteins were dialyzed against PBS (phosphate-buffered saline) to remove imidazole. Lipopolysaccharides (LPSs) were removed from protein solutions using the Toxin Eraser Endotoxin Kit (Genscript, Piscataway, NJ, USA). LPS contamination was detected using an enterobacterial common antigen (ECA) kit (Zhanjiang A&C Biological, Guangdong, China). Concentrations of purified proteins were determined by ultraviolet spectroscopy using an extinction coefficient of 280 nm. Protein solutions were stored at −80°C in PBS.

### *In Vivo* Mouse Procedures and Assays

Macrophages were elicited in mice *via* intraperitoneally injection of 1 ml paroline and harvested from the peritoneal cavity 3–5 days later as described elsewhere (23). Macrophages were cultured in 24-well plates at 5 × 10^5^ cells/ml in DMEM (Hyclone, USA) in a 37°C, 5% CO_2_ atmosphere.

Mice were vaccinated subcutaneously three times at 2-week intervals for 6 weeks with 100 µl PBS containing 8 µg DnaJ and 10 µg ΔA146Ply to ensure a ratio of ΔA146Ply to DnaJ that was identical to that present in an equivalent 18 µg dose of the fusion proteins. PBS injection alone was used as a negative control. DnaJ with 50 µl Al adjuvant (Al PO_4_, Sigma, St. Louis, MO, USA) and the PPV23 (23-valent pneumococcal polysaccharide vaccine, Chengdu Institute of Biological Products, Chengdu, China) were used as positive controls. Serum was collected from mice on the day before immunization for baseline measurements. DnaJ/Ply-specific total IgG and subtyping were determined by indirect ELISA using goat anti-mouse IgG1, IgG2a, IgG2b, and IgG3-HRP conjugates (Santa Cruz, CA, USA).

Splenocytes were isolated from spleens that had been removed from mice 7 days after the last immunization and cultured in 24-well plates at 5 × 10^6^ cells/ml in RPMI 1640 (Hyclone, Barrington, IL, USA) supplemented with 10% fetal bovine serum. Cultured splenocytes were stimulated by adding 5 µg purified protein in PBS and incubated at 37°C in a humidified 5% CO_2_ atmosphere for 3 days. The cytokines IL-4, IFN-γ, IL-10, IL-5, IL-17A, and TNF-α from the culture supernatants were measured using a commercial ELISA kit (Biolegend, San Diego, CA, USA) following the manufacturer’s recommendations.

### Challenge Studies

*Streptococcus pneumoniae* strains were grown on CNA plates with 5% sheep blood in an atmosphere of 5% CO_2_ at 37°C overnight. Bacteria were collected from the agar surface and washed with PBS. Two animal models were started 2 weeks after the last immunization. For the sepsis model, mice received intraperitoneal challenge with a lethal dose of 600 CFU of the pneumococcal strain NCTC7466 (D39, serotype 2). Survival rates of each group were observed and recorded twice daily for 21 days. In colonization model, mice were challenged intranasally with 1 × 10^8^ CFU of the pneumococcal strain CMCC 31693 (serotype 19F), then the bacteria in lung and the nasal wash were collected 72 h later, and suspended in PBS, followed 15 µl of supernatants was plated on blood agar plates with incubation overnight. Lung inflammation of challenged mice was determined by histology. In the colonization model, mice challenged intranasally with 1 × 10^8^ CFU of pneumococcal strain CMCC 31693 (serotype 19F) were sacrificed 72 h later. Lung samples were collected and fixed in 4% paraformaldehyde for 24–48 h, embedded in paraffin, serial sectioned at 5 µm thickness, and stained with hematoxylin and eosin. Tissues were evaluated using light microscopy.

### Passive Immunity

The serum from DnaJ-ΔA146Ply and PBS-vaccinated (sham) mice was collected 2 weeks after the last immunization. Serum samples (200 µl) from immunized and control mice were intraperitoneally transferred to animals immediately before infection with pneumococci (8). The colonization and sepsis models were performed immediately as described above. The bacterial loads of nasal washes and survival rates of mice were measured as described above.

### Intracellular Killing Assays

Macrophages were seeded in 24-well plate at 5 × 10^5 ^cells/well in DMEM (Hyclone, USA) and cultured at 37°C in 5% CO_2_ for 6 h. Anti-DnaJ-ΔA146Ply and non-immune control serum were heat-treated for 30 min in a 56°C water bath to inactivate complement. Pneumococcal strain R6 (5 × 10^6 ^CFU/50^ ^μl) was combined with 50 µl DnaJ-ΔA146Ply-specific serum or non-immune control serum at 37°C in 5% CO_2_ for 15 min for priming. Macrophages were washed five times with PBS and suspended in fresh DMEM at 100 µl per well. The primed pneumococcal strain R6 (100 µl), and sterile serum as a complement source (20 µl), were then added and incubated at 37°C with 5% CO_2_ for 45 min with shaking (220 rpm). Cells were then washed five times with PBS and incubated for 1 h in medium containing penicillin (10 µg/ml) and gentamicin (200 µg/ml) to kill extracellular bacteria. Then, cells were washed five times and lysed with 300 µl of 0.025% saponin-PBS solution for 10 min at room temperature. Bacteria counts were obtained by plating serial dilutions on blood agar plates.

Macrophages were seeded on poly-l-lysine-coated glass coverslips overnight. Pneumococcal strain R6 (OD = 0.5) were washed three times with PBS and then suspended in FITC (8 mg/ml), incubated at room temperature for 30 min in a dark room. Then, the pneumococcal were washed with PBS to discard the redundant FITC, and heat-treated for 30 min in a 65°C water bath to inactivate them. Anti-DnaJ-ΔA146Ply and negative antibodies were also heat-treated for 30 min in a 56°C water bath to inactivate complement at the same time. The primed pneumococcal (100 µl), and sterile baby rabbit serum as a complement source (20 µl), were then added and incubated at 37°C with 5% CO_2_ for 45 min with shaking (220 rpm). After washing with PBS, macrophages on coverslips were incubated with the primed pneumococcal in a dark room for 30 min with shaking. Then, these cells were washed with PBS three times, fixed with 4% PFA for 15 min, and then stained with DAPI for 5 min at room temperature. Cell morphology and fluorescence intensity were observed with a Nikon ECLIPSE 80i microscope (24).

### Statistical Analysis

All data analyses were performed using GraphPad Prism 5 Software (La Jolla, CA, USA). For more than two groups, data with normal distribution and equality of variance were analyzed with one-way variance analysis (ANOVA) simple classification, with Tukey as *post hoc* test to assess statistical significance. And the Kruskal–Wallis non-parametric test was used to analyze the data, even after scale transformation, not normally distributed or without equality of variance. For comparing two independent groups, the survival rates were compared with a log rank test, antibody titers, and numbers of pneumococci (CFU) were analyzed using the Mann–Whitney *U*-test. Cytokines were compared using the Student’s *t*-test or ANOVA followed by Tukey’s test. *P* values of <0.05 were considered to be significantly different.

## Results

### Expression and Purification of Recombinant Proteins

**Figure 1:**
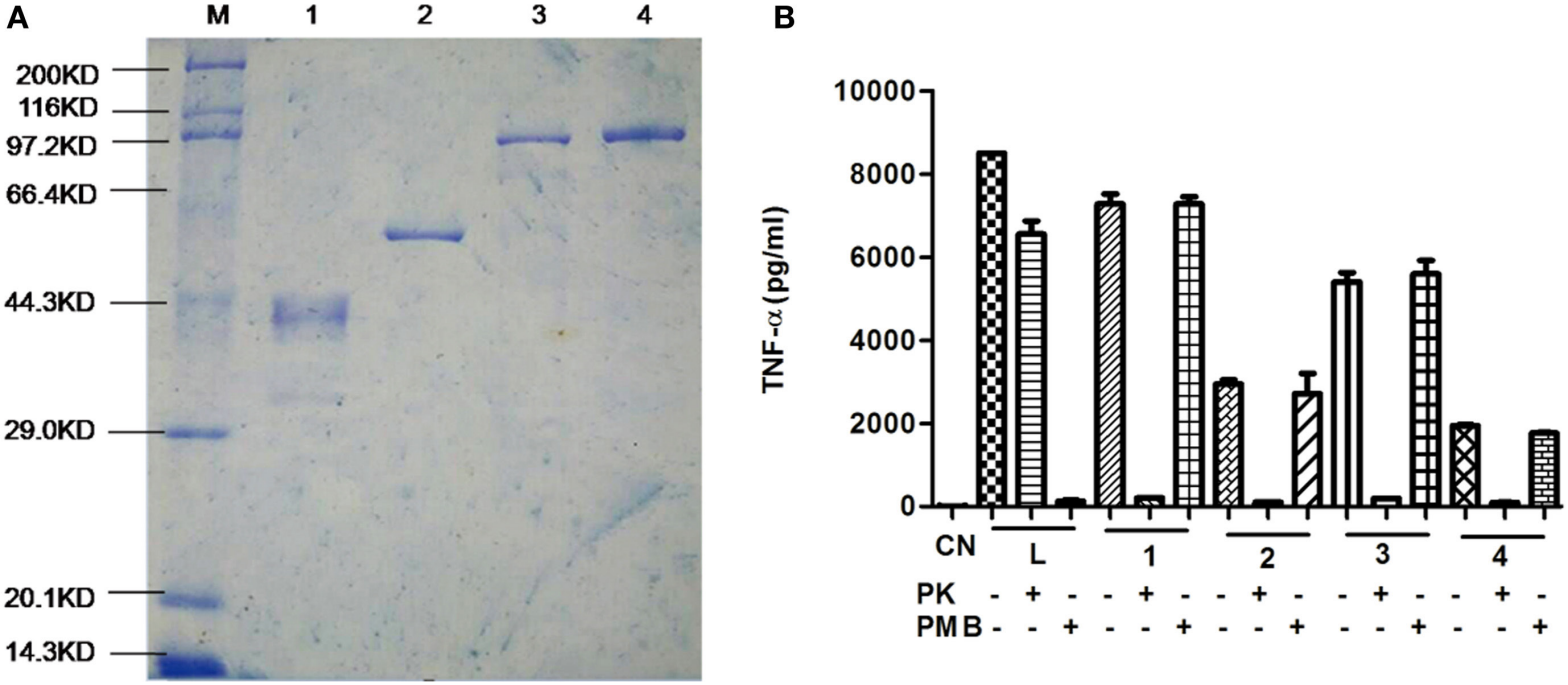
Purification of recombinant proteins. **(A)** Purified proteins were separated using 10% SDS-PAGE and visualized by Coomassie brilliant blue staining. Lane 1. DnaJ, Lane 2, ΔA146Ply, Lane 3, DnaJ-ΔA146Ply, and Lane 4, ΔA146Ply-DnaJ **(B)** ELISA assay for TNF-α expression from macrophages incubated with purified proteins that were pretreated with proteinase K (PK), or polymyxin B (PMB) to remove lipopolysaccharide (LPS). The data are shown as the mean ± SD (*n* = 3). Results are representative of three independent experiments. L, LPS positive control; 1, DnaJ; 2, ΔA146Ply; 3, DnaJ-ΔA146Ply; 4, ΔA146Ply-DnaJ.

We examined our purification of recombinant proteins using SDS-PAGE and each band corresponded with predicted sizes of 38, 50, 93, and 93 kDa, respectively (Figure 1A). Since LPS is a TLR4 agonist, we removed LPS using immobilized polymyxin B columns and assayed the treated proteins for LPS using a commercial ECA kit. Residual LPS levels were below 0.1 EU/μg in our protein preparations. However, to test whether any residual immunogenic LPS remained, purified proteins were incubated with mouse peritoneal macrophages for 24 h and cytokines were assayed using a commercial ELISA kit.

Protein preparations that were pretreated with proteinase K did not stimulate any significant TNF-α expression from macrophages indicating the lack of any significant amounts of residual LPS (Figure 1B). This assay indicated that our protein preparations could be safely evaluated in the following experiments.

### Subcutaneous Administration of the Adjuvant ΔA146Ply Enhances the Production of Antigen-Specific IgG Antibody

**Figure 2:**
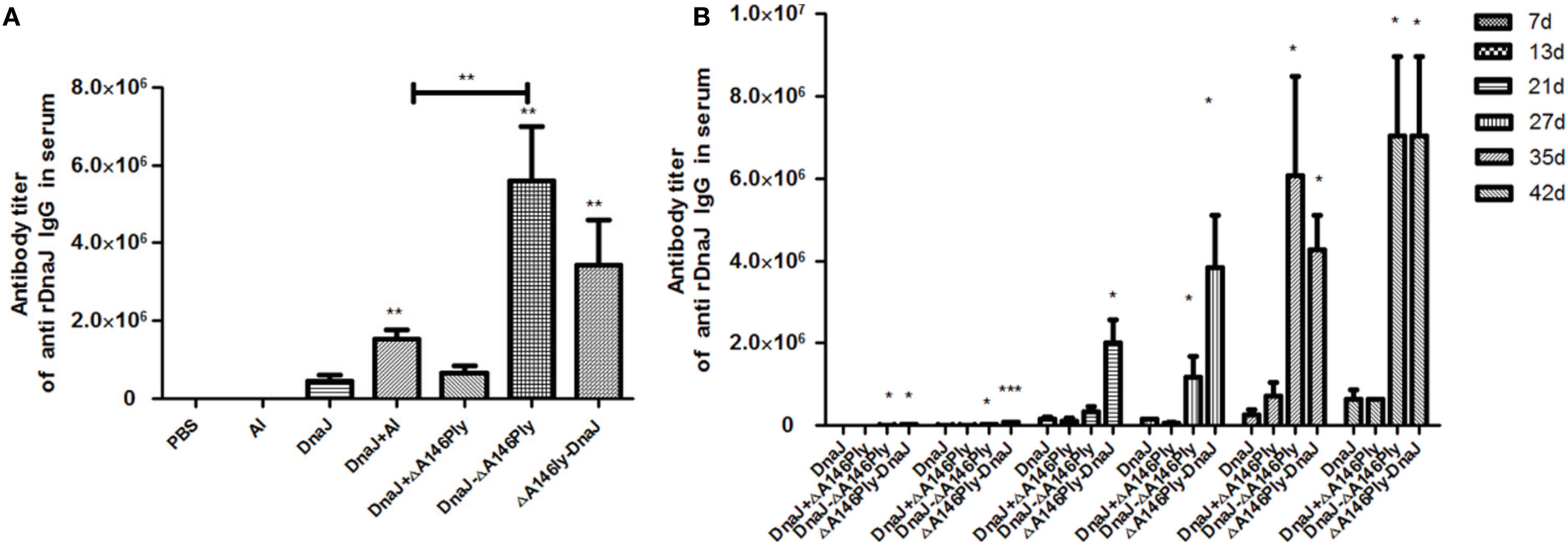
Anti-DnaJ responses in the serum of subcutaneously immunized mice. **(A)** Antibody titers in serum taken from animals immunized with the listed proteins and adjuvants **(B)** Time course of antibody production as a result of vaccination with the agents listed. Data are the mean ± SD and *n* = 4. The results shown are representative of three independent experiments, *P < 0.05 and **P < 0.01 when compared with the DnaJ or DnaJ plus Al group.

We next evaluated whether our purified proteins were immunogenic in mice and measured DnaJ-specific IgG antibodies in serum to evaluate this. Subcutaneous immunization with DnaJ in combination with a commercial Al adjuvant resulted in a small, but significant, increase in anti-DnaJ titers compared with DnaJ alone or in combination with ΔA146Ply. However, both the N-terminal and C-terminal fusion proteins induced titers of DnaJ-specific IgG antibodies to 10^6^–10^7^. This was significantly higher than immunization with DnaJ alone (Figure 2A). Interestingly, the N-terminal fusion protein DnaJ-ΔA146Ply promoted a more rapid and sustained response of anti-DnaJ antibodies. However, by 42 days postimmunization, both fusion proteins had identical titers (Figure 2B).

### Subcutaneous Immunization of Mice with DnaJ-ΔA146Ply Protects against Infection with Pneumococci

**Figure 3:**
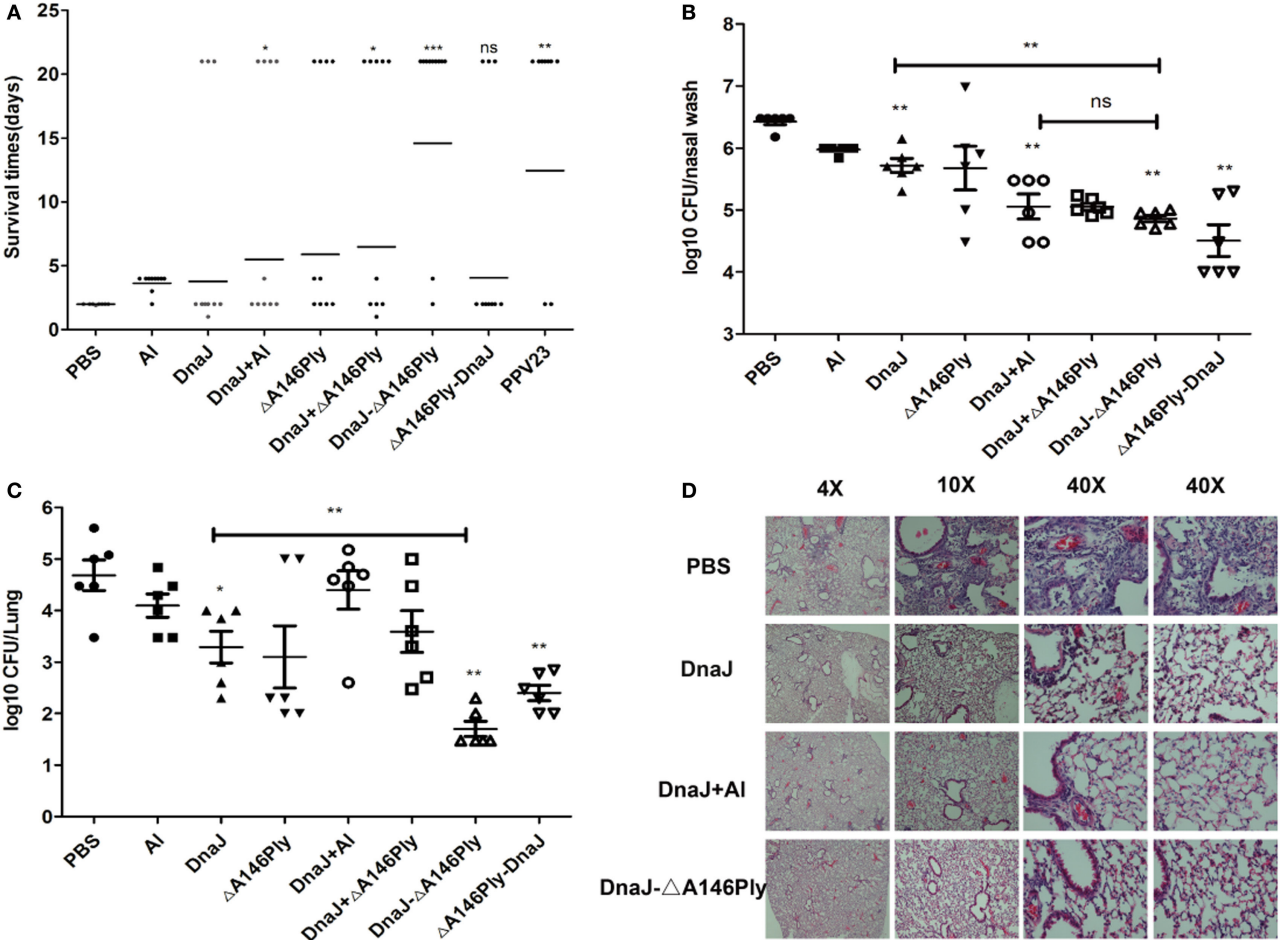
Protective activities of subcutaneous immunization with recombinant proteins. Mice were subcutaneously immunized with the indicated antigens with or without adjuvant and challenged 2 weeks after the last immunization with an i.p. injection of *Streptococcus pneumoniae*. **(A)**. Survival times for immunized mice after a subcutaneously challenge with lethal dose of NCTC7466 (D39, 1,000 CFU). One dot represents one mouse. Each group had 10 C57BL6/J mice. *P < 0.05, **P < 0.01, and ***P < 0.001 when compared with DnaJ group. In colonization model, **(B)** nasopharynx and **(C)** lung colonization of individual mice were determined 72 h after challenged with 19F/CMCC31693 (1 × 10^8 ^CFU). Each group contained 6–8 mice. One square/dot represents one mouse. *P < 0.05, **P < 0.01, and ***P < 0.001 when compared with the PBS group or DnaJ group. **(D)** Pathological analysis of the lung tissues from animals immunized with PBS, DnaJ, DnaJ-ΔA146Ply, and DnaJ plus Al after challenged with 19F (same as above) in the colonization model. The horizontal lines denote the median survival time/bacterial loads for each group. The results shown are representative of three independent experiments.

We next used a pneumococcal sepsis model and immunized C57BL/6 mice were infected with a lethal dose of pneumococcal strain NCTC 7466 (D39, serotype 2) intraperitoneally 2 weeks after the last immunization. Compared to the PBS control group, the survival rates of mice with the N-terminal fusion protein DnaJ-ΔA146Ply alone were significantly lower than when DnaJ and ΔA146Ply were added as separate proteins. The protection rate of DnaJ-ΔA146Ply was 83.3%, which was comparable to that of the polyvalent anti-capsule vaccine PPV23 (Figure 3A). In addition, mice immunized with DnaJ-ΔA146Ply survived significantly longer than mice immunized with DnaJ alone (Figure 3A). This indicated that ΔA146Ply enhances the protection of DnaJ.

In our colonization model, C57BL/6 mice were challenged intranasally with pneumococcal strain CMCC 31693 (serotype 19F) 2 weeks after the last immunization. The bacterial loads from nasal washes and lung homogenates were measured 72 h later. Lung inflammation was detected using fluorescent microscopy.

Subcutaneous immunization with both DnaJ-ΔA146Ply and ΔA146Ply-DnaJ reduced nasal bacterial loads approximately 10–100-fold compared to the PBS control group (*P* < 0.01; Figure 3B). The lung homogenates showed 10–1,000-fold reductions compared to the PBS control group (*P* < 0.01; Figure 3C). We also found that the DnaJ-ΔA146Ply antigen significantly reduced bacterial loads in both the nasal and lung samples compared with DnaJ alone (*P* < 0.01; Figures 3B,C).

The lungs of challenged mice were collected 72 h postinfection and stained with hematoxylin–eosin. Inflammation in the PBS control group was readily apparent with obvious infiltration of inflammatory cells and damage of lung tissue. The DnaJ-ΔA146Ply group fared better with fewer infiltrating cells and normal lung tissue structures (Figure 3D).

Taken together, these results showed that subcutaneous immunization with DnaJ-ΔA146Ply could protect mice against both lethal and sublethal pneumococcal infections. This also indicated that DnaJ-ΔA146Ply is an appropriate candidate for a subcutaneously administered protein vaccine.

### Antibody-Mediated Protection Induced by Subcutaneous Immunization with DnaJ-ΔA146Ply *In Vivo* and *In Vitro*

**Figure 4:**
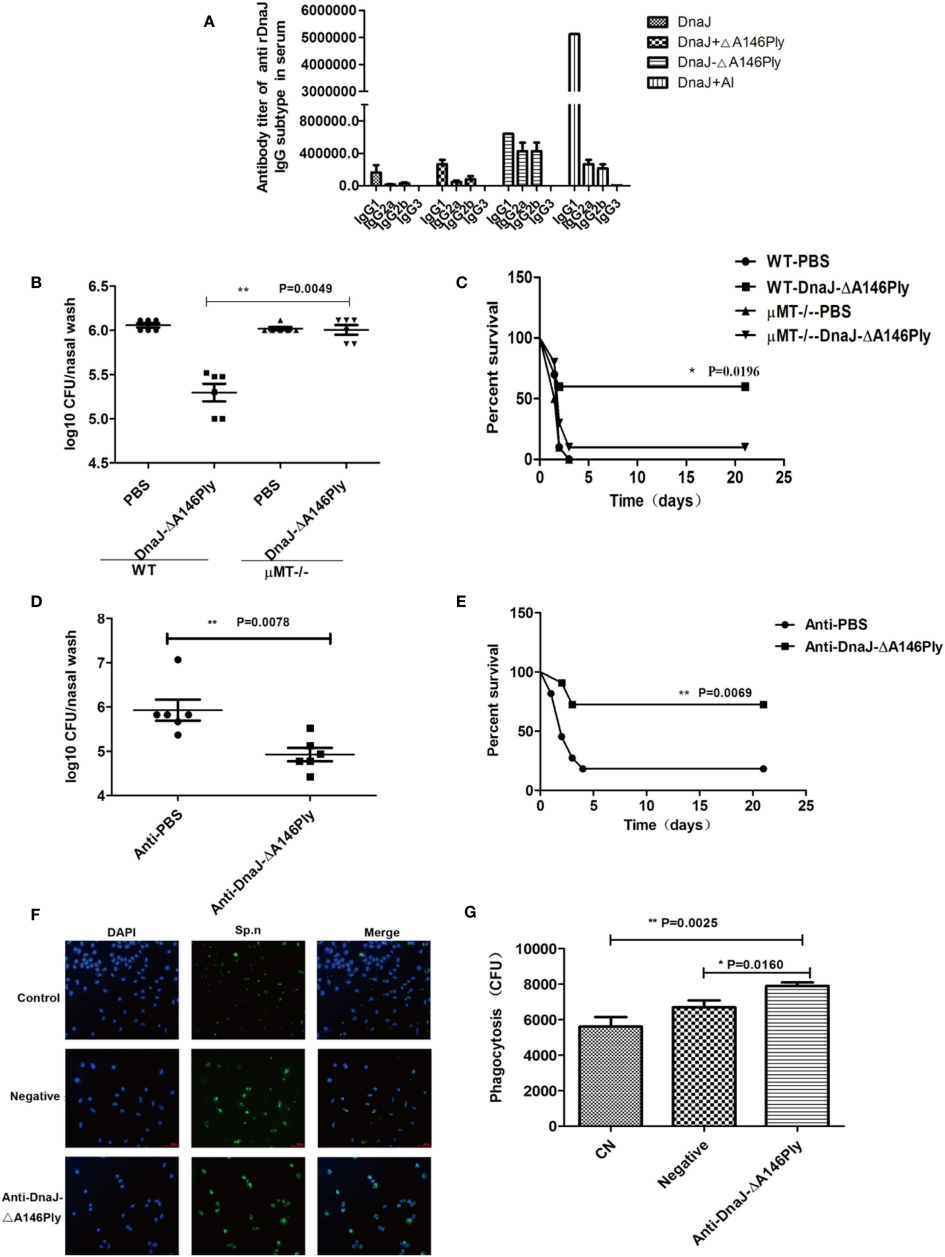
The humoral immune response is involved in the protective effects of subcutaneous immunization with DnaJ-ΔA146Ply *in vivo* and *in vitro*. **(A)** IgG subclasses after subcutaneous immunization of WT mice with purified proteins as listed. Data are the mean ± SD and *n* = 4. **(B,C)** Subcutaneous immunization of μMT and WT mice, mice were immunized with fusion protein DnaJ-ΔA146Ply. **(D,E)** Passive immunization by i.p. injection with 200 µl serum from mice immunized with DnaJ-ΔA146Ply or PBS. Mice were given **(B,C)** intranasally with *Streptococcus pneumoniae* strain CMCC31693 (19F, 1 × 10^8 ^CFU) and **(D,E)** subcutaneously with strain NCTC7466 (D39, 1,000 CFU). Bacterial loads **(B,D)** and survival rates **(C,E)** after intranasal and subcutaneous infection with pneumococcal strains 19F and D39, respectively [*n* = 6 mice/group in **(B,D)**, *n* = 10 mice/group in **(C,E)**]. Antibody-mediated Killing assays of macrophages are shown in **(F,G)**. Killing was defined with photomicrographs in **(F)** and quantified as the CFU counts swallowed in the phagocyte cells in **(G)**. One square/dot represents one mouse. Data are the mean ± SD. The results shown are representative of three independent experiments. *P < 0.05, **P < 0.01.

Mice immunized with DnaJ-ΔA146Ply subcutaneously produced specific IgG antibodies to high titers indicating activation of humoral immunity. We subtyped the IgG antibodies and found IgG1, IgG2a, and IgG2b were equally represented in the DnaJ-ΔA146Ply group. The DnaJ, DnaJ + Al adjuvant and DnaJ + ΔA146Ply possessed the same subtypes but IgG1 dominated (Figure 4A).

We further investigated the role of humoral immunity in protection induced by DnaJ-ΔA146Ply by assessing whether this fusion protein produced sustained immunity. WT and μMT mice immunized with DnaJ-ΔA146Ply and CMCC 31693 (serotype 19F) and NCTC 7466 (D39, serotype 2) were given nasally and subcutaneously, respectively. Significant protection against colonization and lethality were absent in the B-cell deficient μMT mice (Figures 4B–E). These data indicated that the protection by DnaJ-ΔA146Ply vaccination was antibody dependent.

In addition, we passively immunized mice using serum (200 µl) from mice immunized with DnaJ-ΔA146Ply. Serum from non-immunized mice served as a negative control. Mice were then infected with CMCC 31693 (serotype 19F) intranasally or NCTC 7466 (D39, serotype 2) intraperitoneally immediately after serum transfer. The bacterial counts and survival rates indicated that passive immunization significantly enhanced survival rates and reduced bacterial loads compared with negative control serum (Figures 4D,E).

Immune and non-immune serum samples were also used to assess antibody-dependent killing of *pneumococcal* strain R6 by macrophages. DnaJ-ΔA146Ply-specific serum significantly improved the phagocytic killing of bacteria *vs* negative serum (Figures 4F,G). These data suggested that humoral immunity played a key role in the protection given *via* DnaJ-ΔA146Ply immunization.

### Cellular Immunity Involved in the Protection Elicited by Subcutaneous Immunization with DnaJ-ΔA146Ply

**Figure 5:**
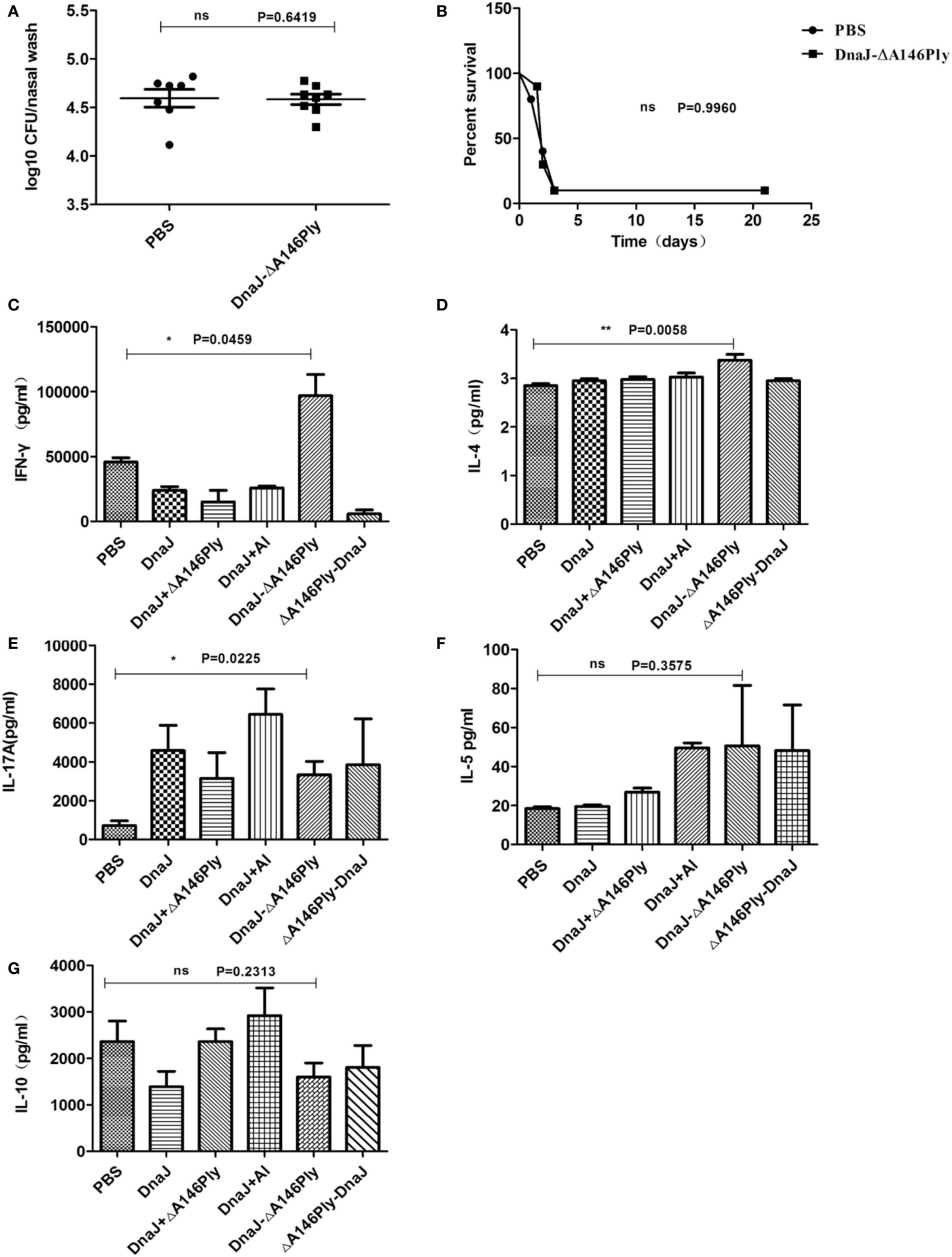
Subcutaneous immunization with DnaJ-ΔA146Ply elicited cellular immune responses. Nude mice and WT mice were subcutaneously immunized with DnaJ-ΔA146Ply and challenged with 19F/CMCC31693 (1 × 10^8 ^CFU) and NCTC7466 (D39, 1,000 CFU) for colonization and lethality models as described above. **(A)** Bacterial loads in nasal washes were determined 72 h postinfection (*n* = 6–8) and **(B)** survival rates were observed until day 21 (*n* = 10). One square/dot represents one mouse. **(C–G)** IFN-γ, IL-4, IL-10, IL-5, and IL-17A produced by splenocytes from immunized mice 7 days after the last immunization (*n* = 3). Data are the mean ± SD. The results shown are representative of three independent experiments. *P < 0.05, **P < 0.01 compared with the PBS group.

Nude mice were used to further explore roles of cellular immunity in protection induced by subcutaneous immunization with DnaJ-ΔA146Ply. The results were similar to those observed in μMT mice. There was a lack of protection after intranasal challenge with 19F as well as with a subcutaneous lethal challenge with strain D39. We found no significant differences between the immunized and PBS control groups (Figures 5A,B).

These data indicated that the immune protection requires both antibody and T cells. Therefore, there is a role for cellular immunity involved in the protection induced by subcutaneous immunization with DnaJ-ΔA146Ply. However, we did not know the T-cell subtype (Th1, Th2, Th17, or Treg) was involved in the process. We therefore measured levels of IFN-γ, IL-4, IL-5, IL-10, and IL-17A from splenocytes by ELISA.

Spleen cells from mice immunized with DnaJ-ΔA146Ply produced significantly higher amounts of IFN-γ and IL-17A than control mice (Figures 5C–E), but there was no difference in the amounts of IL-5 and IL-10 between the immunized groups and controls (Figures 5F,G). This suggested that Th1 and Th17 cellular responses were activated *via* subcutaneous immunization with DnaJ-ΔA146Ply.

Although the level of IL-4 was low, there was significant difference between DnaJ-ΔA146Ply group and PBS control (*P* = 0.0058; Figure 5D). In order to more precisely determine the contributions of cytokine IL-4, Th1, and Th17 cells, we immunized IL-4, IFN-γ, and IL-17A-deficient mice with DnaJ-ΔA146Ply or PBS, and WT mice were immunized as controls. We then tested immune protection using the nasal colonization and sepsis models as described above.

**Figure 6:**
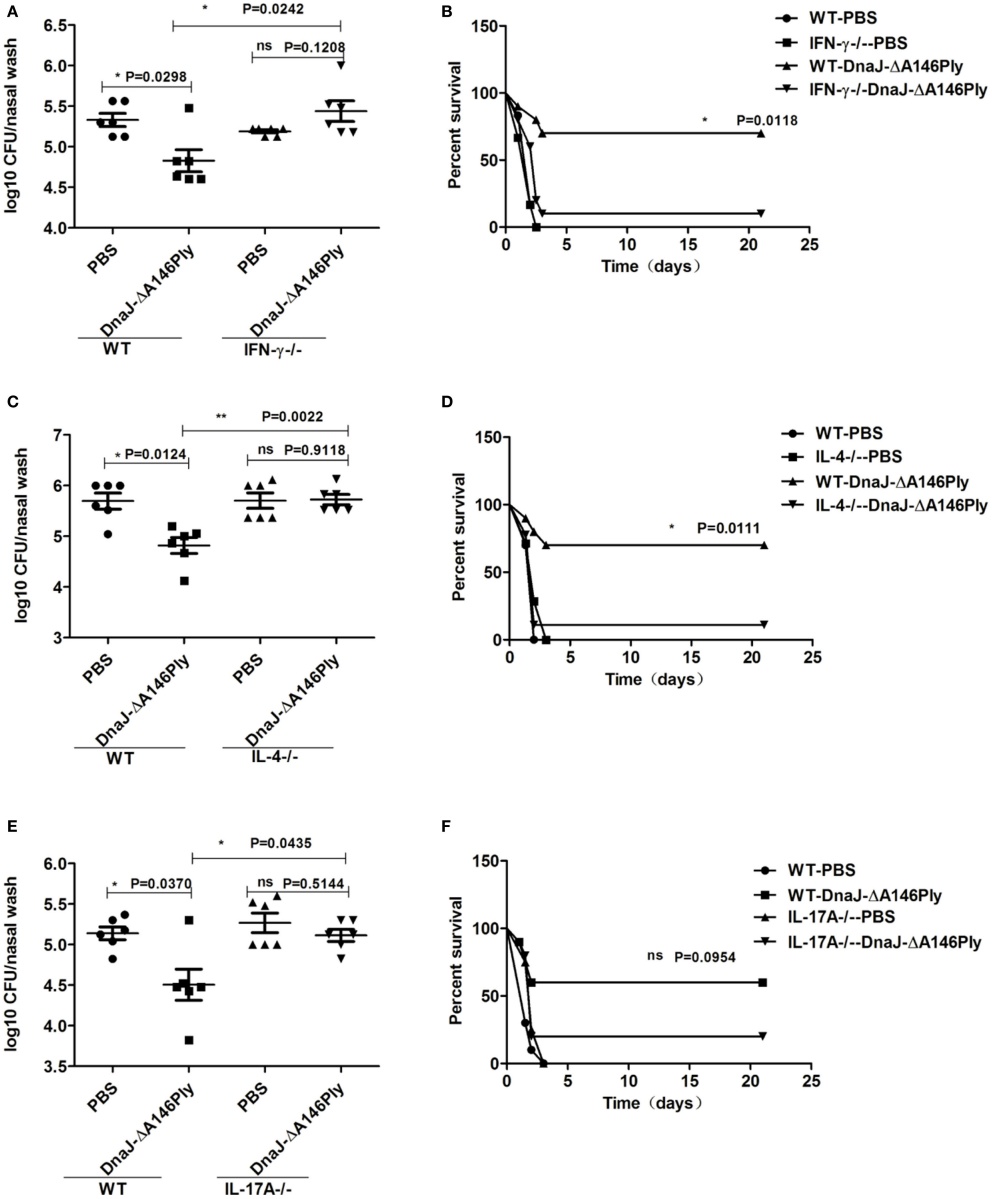
Th1, Th17 cellular immunity, and cytokine IL-4 are vital for the protective effects induced by subcutaneous immunization with DnaJ-ΔA146Ply. Knockout mice and WT mice were immunized with DnaJ-ΔA146Ply and challenged with 19F/CMCC31693 (1 × 10^8 ^CFU) and NCTC7466 (D39, 1,000 CFU) to set up the colonization model and sepsis models, respectively. **(A,C,E)** Bacterial loads in nasal washes were detected 72 h postinfection (*n* = 6) and **(B,D,F)** survival were observed until day 21 (*n* = 10). One square/dot/triangle represents one mouse. Data are the mean ± SD. The results shown are representative of three independent experiments. *P < 0.05, **P < 0.01; ns, not significant; IFN-γ^−/−^, IFN-γ-deficient mice; IL-4^−/−^, IL-4-deficient mice; IL-17A^−/−^, IL-17A-deficient mice.

In IFN-γ^−/−^ mice, the survival rate was decreased (*P* = 0.0118; Figure 6A), and the nasal bacterial load was much higher than that in WT mice (*P* = 0.0242; Figure 6B). Similar results were obtained in IL-4-deficient mice (*P* = 0.0111, *P* = 0.0022; Figures 6C,D). However, in IL-17A-deficient mice, the colonization of CMCC 31693 (serotype 19F) was significantly attenuated compared with that of WT mice (*P* = 0.0435; Figure 6E). The protection against lethal infection of NCTC 7466 (D39, serotype 2) was reduced to a degree, but the differences were not significant (*P* = 0.5144; Figure 6F).

Together, these data indicated that cellular immunity is involved in protection elicited by subcutaneous immunization with DnaJ-ΔA146Ply. Th1, Th17 cellular immune responses, and cytokine IL-4 were responsible for the protection of DnaJ-ΔA146Ply against the colonization of pneumococcus in the upper respiratory tract. Th1 cell and cytokine IL-4 also participated in the protection of DnaJ-ΔA146Ply against pneumococcus lethal infection.

### TLR4 is Involved in Vaccine-Mediated Bacterial Clearance and Immune Response Stimulation

The Ply protein is a TLR4 ligand and DnaJ can elicit a TLR4-dependent macrophage response (21). To investigate whether TLR4 also plays an important role in protection induced by subcutaneous immunization with DnaJ-ΔA146Ply, TLR4-deficient mice were immunized with DnaJ-ΔA146Ply to observe whether it can influence immune response activation and protection.

**Figure 7:**
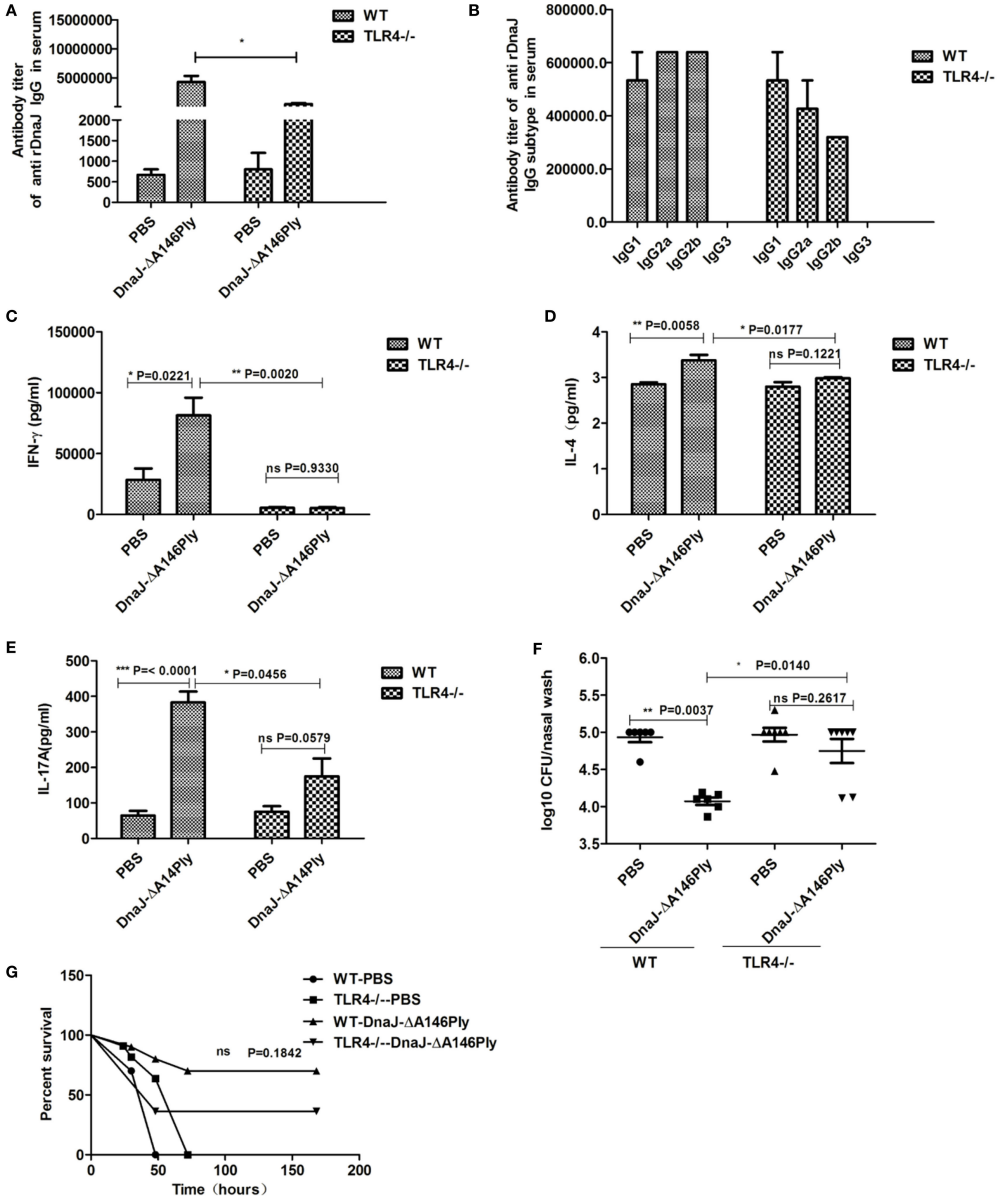
TLR4 participates in vaccine-mediated bacterial clearance and the stimulation of the immune response. TLR4-deficient mice and WT mice were subcutaneously immunized with DnaJ-ΔA146Ply. Seven days after the last immunization, the **(A)** titer and **(B)** subclass of DnaJ-specific antibodies in serum from immunized mice were identified by ELISA (*n* = 4). Splenocytes cultured from mice were assayed for the levels of cytokines **(C)** IFN-γ, **(D)** IL-4, and **(E)** IL-17A (*n* = 3). Mice were challenged with 19F/CMCC31693 (1 × 10^8 ^CFU) and NCTC7466 (D39, 1,000 CFU) as described in the text. **(F)** Bacterial loads in nasal washes (*n* = 6–8) and **(G)** survival rates (*n* = 10). Data are the mean ± SD. The results shown are representative of three independent experiments. *P < 0.05, **P < 0.01. TLR4^−/−^, TLR4-deficient mice.

In TLR4-deficient mice, specific antibody titers in serum were significantly lower than that in WT mice (Figure 7A). However, the subtypes were similar to those in WT mice but at lower levels (Figure 7B). Compared to WT mice, the levels of protective cytokines IFN-γ, IL-4, and IL-17A in splenocytes had decreased significantly in TLR4-deficient mice immunized subcutaneously with DnaJ-ΔA146Ply (Figures 7C–E). Interestingly, there was a significant difference between the TLR4-deficient mice and WT mice immunized subcutaneously with DnaJ-ΔA146Ply in nasal bacterial counts (*P* = 0.014; Figure 7F). However, these mice still showed a 40% survival rate when vaccinated TLR4-deficient mice (*P* = 0.1842; Figure 7G).

Together, these data suggested that TLR4 affects DnaJ-ΔA146Ply-mediated bacterial clearance in the upper respiratory tract. However, TLR4-deficient mice immunized with DnaJ-ΔA146Ply were provided a certain amount of protection against pneumococcus lethal infection. This may be the result of other pathways that we did not investigate in this study.

## Discussion

Subcutaneous immunization is widely used in clinics because it is simple and rapid. A protein vaccine with adjuvant immunization can be effective against *S. pneumoniae* infection after subcutaneous immunization (8, 25). Although a variety of subcutaneous immunization protein vaccines have been developed, there is a need for adjuvants to produce the optimal immune effect. The adjuvant currently used for humans is primarily Al (26). Al adjuvant is weak CTL-responses and also can cause some adverse reactions. For example, inoculation of aluminum adjuvant vaccine can cause local adverse reactions such as erythema, subcutaneous nodules, contact hypersensitivity, and granuloma (26, 27). A new, safe subcutaneous immune adjuvant is needed and is thus a major focus of vaccine research. A variety of compounds with adjuvant properties currently exist, such as mineral salts, saponins, cytokines, and liposomes (28). There are several licensed adjuvants for humans such as MF59, AS03/04 and R837/848 (29), but TLR4 ligands are also promising. For example, TLR4 agonist adjuvant MPL is a component of the licensed HPV vaccine Cervarix (30). Our previous studies showed that a TLR4 agonist ΔA146Ply (non-hemolytic) can function as an effective mucosal adjuvant (10). In this study, we demonstrated that ΔA146Ply can also be used as a subcutaneous immune adjuvant.

In the current study, we used DnaJ-ΔA146Ply for subcutaneous immunization of mice and found it to be effective against the colonization of *S. pneumoniae* in the nasopharynx, but it also reduced pulmonary inflammation. The protective effect of DnaJ-ΔA146Ply was better or equal to the DnaJ plus Al group and had an 83.3% survival rate from lethal infection. In addition, it performed as well as the multivalent anticapsule vaccine, PPV23. These results indicate that ΔA146Ply can assist DnaJ to stimulate a rapid antibody response and increase antibody and cytokine levels against lethal infection. ΔA146Ply has certain subcutaneous immunization adjuvant effect.

A high-quality protein vaccine can induce humoral and cellular immune responses simultaneously with a strong protective effect (31). We evaluated these two aspects of humoral and cellular immune responses induced by DnaJ-ΔA146Ply subcutaneous immunization. First, it stimulated a high titer of specific antibody indicating humoral immune activation. Second, in B cell-deficient μMT mice, the protective effect was eliminated providing further evidence for the key role of humoral immunity. Antibody-mediated phagocytosis in macrophages was also stimulated with DnaJ-ΔA146Ply antiserum. Passive protection was also successful confirming that the antiserum was effective against colonization of *S. pneumoniae* in the nasopharynx as well as against lethal infection.

A lack of colonization protection was also seen following intranasal vaccination with the live attenuated vaccine SPY1 in μMT mice (15). Additionally, a similar need for humoral immunity against colonization was identified in immune protection elicited by a non-encapsulated pneumococcus strain, but not in the protection induced by an encapsulated strain (32, 33). Thus, B cells may be needed to produce specific antibody to participate in the protection against pneumococcal infections.

When we used T cell-deficient mice, both colonization and survival rates were significantly lower, even with immunization. This suggested that T cells play an important role in the protective effect induced by DnaJ-ΔA146Ply. This has been previously shown for Th1 and Th17 cells (31). We also found that IFN-γ and IL-17A levels were significantly higher in splenocytes from immunized mice indicating that Th1 and Th17 cells were activated. Therefore, mucosal immunity with the fusion protein only exerted a significant increase in IL-17A while subcutaneous immunity induced much wider immune effects.

A number of studies have shown that the Th1 immune response plays an important role in the protective effects of *S. pneumoniae* vaccines against fatal infections (34). IFN-γ is Th1 cytokine and inactivating IFN-γ decreases survival rates after *S. pneumoniae* challenge while IFN-γ treatments significantly increased survival rates (35). These results suggest that Th1 cells are key players in *S. pneumoniae* immunity.

The high titers of IgG2a/b antibodies and concentration of IFN-γ in our study suggested that DnaJ-ΔA146Ply induced a strong Th1 response. Additionally, we found that not only mouse survival rates but also nasopharyngeal colonization of pneumococci in IFN-γ-deficient mice significantly decreased compared with WT mice. This demonstrates that the protective effect of DnaJ-ΔA146Ply regarding lethal challenge and colonization of mice relies on Th1 cells.

Our results found that the concentration of IL-4 in fusion protein group was higher than that of PBS control, and then, we proved that IL-4 was very important in the protection mediated by DnaJ-ΔA146Ply. However, the level of IL-4 was low, indicating that it might not be secreted by typical Th2 subset. As we know, follicular helper T cells also produce low levels of IL-4. However, our results (data not shown) showed that there was no difference in antibody production between WT and IL-4^−/−^ mice. As an immune cell that provides a helper function to B cells, follicular helper T cells might not be the source of IL-4 in our study either. It needs further research to identify which subset of IL-4-secreting T cells takes part in the protection against *S. pneumoniae* infection in our study.

Th17 cells also participate in *S. pneumoniae* infection clearance and inhibition of nasal recolonization by secreting IL-17 (10, 31). Th17 cells can also resist bacterial colonization by enhancing neutrophil infiltration, increasing antibacterial peptide production from epithelial cells, and enhancing the integrity of the epithelial layer (36, 37). IL-17A has also been implicated in the antibody response to *S. pneumoniae* capsular polysaccharide and can effectively enhance bacterial phagocytosis and killing functions to resist lethal infection (38). However, there is controversy over whether IL-17A is involved in resisting *S. pneumoniae* lethal infections.

In our study, the results using IL-17A-deficient mice indicated that Th17 cells are primarily involved in vaccine-mediated clearance of *S. pneumoniae* from the nasopharynx. However, resistance to lethal infections did not differ from WT mice. The IL-17A in immune WT mice may enhance neutrophil and macrophage infiltration, effectively eliminating the infection in the nasopharynx (15).

Our data indicated that both humoral immunity and cellular immunity are involved in the protection elicited by subcutaneous immunization with DnaJ-ΔA146Ply. These results differed from a mucosal immunization route with this protein because Th17, Th1 cells, and cytokines IL-4 all played important roles in protection against colonization and lethal infection. Interestingly, the C-terminal fusion protein ΔA146Ply-DnaJ could not protect against lethal infection although did function in against nasopharyngeal colonization. These results are consistent with results of mucosal immunity (10). This may indicate that the immune activation structure of fusion proteins for subcutaneous immunity may be same as that for mucosal immunity. But these hypotheses still need experimental verification.

The DnaJ and Ply proteins have been shown to participate in TLR4-dependent immune responses (20, 21). Therefore, we initially hypothesized that TLR4 may be needed for the use of DnaJ-ΔA146Ply as a vaccine. When we immunized TLR4-deficient mice with DnaJ-ΔA146Ply, specific antibodies and cytokines declined and nasopharyngeal bacteria were not cleared. This indicated that TLR4 is involved in the activation of mice immune system by DnaJ-ΔA146Ply. Adaptive immunity was initiated to promote the production of antibodies by B cells and cytokines by CD4+ T cells. This process would thereby promote bacterial clearance and resist infections in the nasopharynx.

However, it was surprising that there were no significant differences between the survival rate of TLR4-deficient mice and WT mice. Therefore, in the TLR4-deficient mouse septicemia model, there may be other receptors involved, so that the vaccine can still induce a certain protective effect. Ply immunization can induce NLRP3 inflammasome activation to enable TLR4-independent IL-1β expression. This would contribute to host protection against *S. pneumoniae* infection (39, 40). However, neither DnaJ-ΔA146Ply nor ΔA146Ply could induce the secretion of IL-1β in the present study (data not shown). This suggested that NLRP3 was not a participant in the protective effects of subcutaneous immunization with DnaJ-ΔA146Ply.

Other studies have shown that in the early stages of *S. pneumoniae* infection, TLR9 activates pulmonary macrophage phagocytosis to reduce the amount of controlled bacterial loads. However, TLR9 did not participate in the control of bacterial colonization of the nasopharynx (41). Scavenger receptors expressed by splenic marginal zone macrophages are involved in *S. pneumoniae* clearance in a septicemia model (42–44). But further experiments are needed to identify the exact receptors and pathways activated by the DnaJ-ΔA146Ply vaccine.

In summary, our study shows that ΔA146Ply has a subcutaneous immune adjuvant effect. Mice immunized with DnaJ-ΔA146Ply *via* this route could be resistant to *S. pneumoniae* infection by inducing both humoral and cellular immune responses. Protection against colonization also depends on TLR4.

## Ethics Statement

All animal experiments were carried out in accordance with the recommendations of the Institutional Animal Care and Use Committee of Chongqing Medical University. Experimental protocols involving animals were approved by the Animal Ethics and Research Committee of China University Graduate School of Medicine.

## Author Contributions

YS, DL, YY, and XZ performed experiments and participated in the design of the study; YX, HW, and XW performed experiments; FC and JY performed statistical analysis; YS, DL, JW, and XZ wrote the manuscript. All authors have read and approved this version of the article, and due care has been taken to ensure the integrity of the work.

## Conflict of Interest Statement

The authors declare that the research was conducted in the absence of any commercial or financial relationships that could be construed as a potential conflict of interest.
